# Hearing Loss, Cognitive Decline, and Dementia: Clinical Intersections

**DOI:** 10.3390/audiolres16040097

**Published:** 2026-06-30

**Authors:** Danielle S. Powell, Carrie L. Nieman, Per Thorsell, Natalie A. Phillips, Ingrid Ekström

**Affiliations:** 1 Department of Hearing and Speech Sciences, University of Maryland, College Park, MD 20742, USA; dspowell@umd.edu; 2 University of Maryland Institute for Health Computing, North Bethesda, MD 20852, USA; 3 Center for Seniors Uniting Nationwide to Support Health Integrated Care and Economics, University of Maryland, College Park, MD 20742, USA; 4 Department of Otolaryngology-Head & Neck Surgery, Johns Hopkins School of Medicine, Baltimore, MD 21205, USA; cnieman1@jhmi.edu; 5 Johns Hopkins Cochlear Center for Hearing & Public Health, Johns Hopkins Bloomberg School of Public Health, Baltimore, MD 21205, USA; 6 Aging Research Center, Department of Neurobiology, Care Sciences and Society, Karolinska Institutet, Stockholm University, 114 19 Stockholm, Sweden; per.thorsell@ki.se; 7 Department of Psychology, Concordia University, Montreal, QC H3G 1M8, Canada; natalie.phillips@concordia.ca

**Keywords:** hearing impairment, cognition, Alzheimer’s disease, cognitive aging, sensory health

## Abstract

**Background/Objectives**: Age-related hearing loss is one of the most prevalent chronic conditions in older adults and has emerged as a potentially modifiable risk factor for cognitive decline and dementia. Increasing evidence from epidemiological, neurobiological, and interventional studies has improved our understanding of the complex relationships between auditory dysfunction and cognitive aging. By highlighting findings in these areas, this review aims to aid clinicians and researchers gain a better understanding of the association between hearing loss, cognitive decline and dementia, and the importance of considering sensory decline during cognitive screening. **Methods**: This narrative review summarizes and integrates findings from epidemiological, neurobiological, and clinical studies examining relationships between hearing loss, cognitive decline, and dementia. Particular focus was placed on epidemiological associations, proposed mechanistic pathways, implications for screening and diagnostic assessment, and evidence regarding hearing rehabilitation interventions. **Results**: Accumulating evidence indicates that hearing loss is associated with accelerated cognitive decline and increased dementia risk. Proposed mechanisms include increased cognitive load, reduced sensory input, social isolation, depression, and shared neurodegenerative or vascular pathology, although causal pathways remain incompletely understood. Emerging evidence suggests that hearing rehabilitation may help preserve cognitive function in some groups, but findings remain heterogeneous. Clinical studies further support the importance of considering auditory function during cognitive assessment, as unrecognized hearing impairment may influence test performance, communication, and diagnostic accuracy. **Conclusions**: Current evidence supports hearing loss as an important factor in cognitive aging and dementia research and highlights the potential value of integrating hearing assessment and management into clinical and research settings.

## 1. Introduction

Hearing plays a fundamental role in social interaction and engagement with the surrounding environment, suggesting multiple pathways through which hearing health may influence cognitive aging and brain health. Over the past two decades, growing evidence has linked hearing impairment to cognitive decline, mild cognitive impairment, and dementia. These observations have generated considerable interest in hearing loss within dementia research and prevention. Large population-based studies have consistently reported associations between hearing impairment and accelerated cognitive decline as well as increased risk of incident dementia [1,2,3]. Consequently, hearing loss has been incorporated into current dementia prevention frameworks as a potentially modifiable risk factor for cognitive decline or dementia [4].

Yet, important questions remain regarding the nature of the relationship between hearing impairment, cognitive decline, and dementia, and the implications of incorporating hearing health considerations into clinical practice and dementia prevention strategies. Important questions remain regarding the role of hearing loss in cognitive aging, including whether it acts as a causal contributor or an early marker of neurodegeneration, whether treatment can modify cognitive trajectories, and how hearing assessment and management can be effectively integrated into clinical care and dementia prevention strategies.

These questions may be most productively examined through a prevention-oriented and life-course perspective where hearing health is considered not only in relation to individuals with established dementia, but also as part of broader strategies aimed at maintaining cognitive health across aging [3]. Within such an approach, hearing loss may play different roles across stages of cognitive aging: as a potentially modifiable risk factor in primary prevention, as an early clinical indicator of vulnerability in secondary prevention, and as a contributor to functional decline among individuals with emerging cognitive impairment. Together, this perspective highlights hearing health as a relevant target across the full continuum of cognitive aging, with implications for both research and clinical practice.

Several recent reviews have summarized the rapidly expanding literature on hearing loss and cognition. Powell and colleagues [5] provide an overview of epidemiological findings linking hearing impairment with cognitive decline and dementia, as well as proposed mechanisms underlying this association. Subsequent work has further explored whether hearing rehabilitation could influence cognitive outcomes, potentially through both direct neural mechanisms and indirect pathways such as improved communication and social engagement [6].

Building on this body of work, this narrative review was developed by a multidisciplinary team based in Sweden, Canada, and the United States, bringing together expertise in audiology, hearing rehabilitation, epidemiology, psychophysics, neuropsychology, cognitive aging, and dementia. It aims to provide an accessible overview of current knowledge on the relationship between hearing loss, cognitive decline, and dementia, with particular attention to implications for audiology practice. Specifically, we summarize epidemiological evidence linking hearing impairment to cognitive outcomes, outline proposed mechanisms connecting auditory and cognitive aging, and review findings on hearing rehabilitation and cognitive trajectories. In contrast to prior reviews, we take a prevention-oriented, life-course perspective, emphasizing how hearing loss may play distinct roles across stages of cognitive aging and how this can inform clinical practice. By integrating evidence from population-based, experimental, and clinical studies, we aim to move the field beyond description and take steps toward identifying what can reasonably be concluded, what remains uncertain, and where intervention and implementation efforts may be most impactful.

## 2. Methods

This narrative review aimed at summarizing current evidence on the relationships between hearing loss, cognitive decline, and dementia. Relevant literature was identified through searches conducted independently by two authors (I.E. and P.T.) using PubMed, Google Scholar, and Karolinska Institutet Library resources. Search terms included combinations of hearing loss, hearing impairment, cognition, cognitive decline, dementia, Alzheimer’s disease, hearing aids, hearing rehabilitation, and related terms. The initial literature search was subsequently complemented by the remaining authors based on their respective areas of expertise. Additional articles were identified through reference lists of key publications.

Studies were selected based on their relevance to the review objectives. When interpreting findings, greater weight was given to evidence from large population-based cohort studies, randomized controlled trials, and high-quality systematic reviews and meta-analyses. Studies with larger sample sizes, audiometric assessments, standardized cognitive or dementia outcomes, and appropriate adjustment for major confounders were prioritized over smaller or methodologically limited studies. Risk of bias was considered qualitatively during interpretation, including issues such as residual confounding, selection bias, reliability and validity of hearing and cognitive measures, and attrition in longitudinal cohorts.

As this work was designed as a narrative review rather than a systematic review, formal study screening procedures, predefined inclusion and exclusion criteria, and quantitative risk-of-bias assessments were not performed. The review should therefore be interpreted as a narrative synthesis of the literature rather than a systematic evaluation of all available evidence.

## 3. Epidemiological Evidence Linking Hearing Loss and Cognitive Decline

Population-based studies can be used to examine the health trends, patterns or risks of large groups of individuals to inform clinical practice guidelines and disease prevention strategies. A wide variety of studies have sought to understand the relationship between hearing impairment and cognitive aging, examining whether hearing loss is associated with lower cognitive performance, accelerated cognitive decline, and/or increased risk of dementia. These studies also offer insight into the magnitude of associations, potential time windows of risk, and the extent to which hearing impairment may overlap with or independently predict cognitive outcomes. Importantly, several studies have shown that hearing loss is associated with poorer performance and more pronounced decline even on non-auditory cognitive tests, suggesting that these associations cannot be fully explained by difficulties in perceiving or encoding auditory information alone [7]. Although observational evidence cannot establish causality, consistent patterns across studies suggest hearing loss may represent both a potential risk factor for cognitive decline and an early indicator of broader neurobiological vulnerability.

Table 1 gives an overview of available population-based studies investigating the association between hearing impairment and cognitive function or dementia. In summary, cross-sectional analyses within large cohort studies have consistently demonstrated lower cognitive performance among individuals with hearing loss, including on predominantly non-auditory cognitive tasks, even after adjustment for age, sex, education, and multiple health-related risk factors [1,8,9,10,11]. Longitudinal cohort studies provide stronger evidence regarding the temporal relationship between hearing impairment and cognitive outcomes and better capture how hearing loss may influence changes in cognition over time. Across multiple large-scale longitudinal studies, baseline hearing loss has been associated with accelerated cognitive decline and increased risk of dementia [8,10,12,13,14,15].

### 3.1. Interpretational Considerations

While longitudinal studies showing that hearing loss precedes incident cognitive decline and dementia are informative, findings from observational studies should still be interpreted with some caution. Associations may, at least in part, reflect shared underlying factors not captured in the study analyses rather than direct causal effects. For example, socioeconomic conditions, cardiovascular health, social engagement, and access to healthcare may influence both hearing and cognitive outcomes but are not always comprehensively captured across cohorts [16,17,18,19,20,21]. If not adequately captured, estimates of risk or association may be elevated or reduced. Differences in hearing loss definitions, cognitive and dementia criteria, follow-up periods, and participant characteristics may contribute to heterogeneity in reported effect sizes, making it more difficult to compare findings and draw consistent conclusions regarding the relationship between hearing loss and cognitive decline [6]. In addition, publication bias may influence the available literature, as studies reporting statistically significant associations between hearing loss and cognitive outcomes may be more likely to be published than studies reporting null findings. This possibility should be considered when interpreting the overall strength and consistency of the evidence.

### 3.2. Hearing Loss as a Target for Dementia Prevention

The growing body of epidemiological evidence linking hearing impairment to cognitive decline has led to increasing interest in hearing loss within dementia prevention frameworks. The Lancet Commission identified hearing loss as one of the most important modifiable contributors to dementia risk in the population, in large part because it is very common—globally, approximately 1 in 5 individuals are estimated to have at least mild hearing loss [22]—and can be treated. Therefore, addressing hearing loss could potentially reduce the number of dementia cases at the population level [3,23]. Similar conclusions have been drawn in subsequent systematic reviews examining hearing impairment within global dementia risk models [24].

However, these estimates depend on assumptions regarding partial causal relationships between hearing impairment and dementia. As a result, interpretations for clinical utilization should be cautious as these estimates are intended for the population level and are not representative of individual risk. Although hearing loss represents a promising target for prevention strategies due to its high prevalence and potential treatability, uncertainties remain regarding the extent to which treatment of hearing impairment can directly alter long-term cognitive trajectories [25]. These uncertainties are addressed in a later section of this paper (see Section 6, Hearing Rehabilitation and Cognitive Outcomes).

Taken together, epidemiological research consistently demonstrates associations between hearing loss and adverse cognitive outcomes across cross-sectional and longitudinal designs. These findings have elevated hearing health as an important consideration in dementia research and prevention, while also highlighting the need for mechanistic studies and intervention trials to clarify causal pathways.

## 4. Mechanisms Linking Hearing Loss, Cognitive Decline, and Dementia

**Figure 1:**
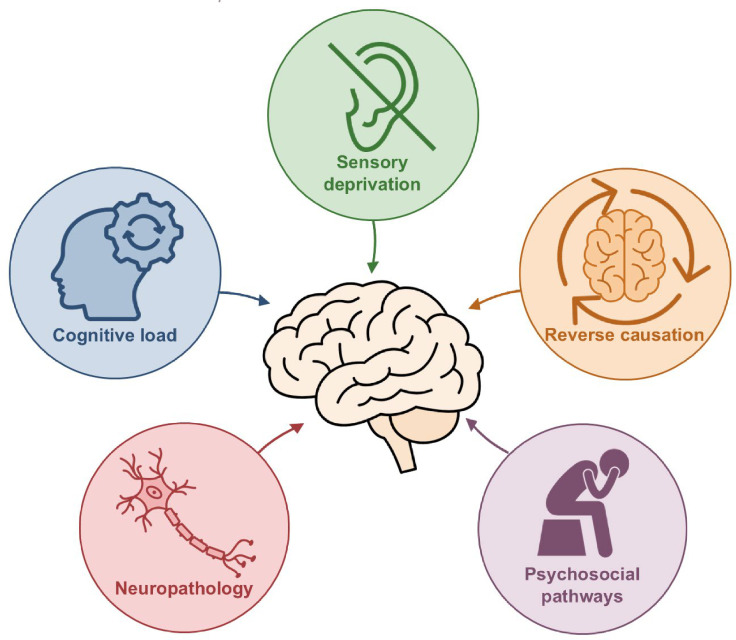
Mechanisms proposed to explain the link between hearing loss and cognitive decline, including cognitive load, sensory deprivation, psychosocial pathways, shared neuropathology, and reverse causation. These pathways are not mutually exclusive and may interact across aging.

Several mechanisms have been proposed to explain the association between age-related hearing loss and cognitive decline. Clarifying the underlying pathways informs clinical practice, as it determines whether interventions targeting hearing loss are likely to influence cognitive trajectories, serve as indicators of underlying disease, and shapes perceptions of how hearing loss fits into other disciplines’ strategies for dementia prevention. These pathways are not mutually exclusive and likely interact across the course of aging. Broadly, five main, partially overlapping mechanisms have been proposed, namely, increased cognitive load and resource allocation, sensory deprivation and neuroplasticity, psychosocial pathways involving social isolation and depression, shared neuropathological processes, and reverse causation, in relation to early neurodegenerative changes affecting central auditory processing (Figure 1).

### 4.1. Cognitive Load and Resource Allocation

One prominent explanation is the cognitive load or effortfulness hypothesis. As auditory signals become harder to decode with hearing loss, more cognitive resources must be allocated to speech perception, leaving fewer available for memory and other higher-order cognitive processes [26,27]. Behavioral studies support this account, showing that hearing impairment increases listening effort and can impair performance on concurrent cognitive tasks [28]. The Ease of Language Understanding (ELU) model similarly proposes that degraded input disrupts automatic speech processing and instead requires explicit working-memory and executive support [29]. Neuroimaging findings converge with this view. Greater hearing loss has been associated with stronger connectivity between auditory cortex and the cingulo-opercular network [30], and with increased frontal recruitment during challenging listening conditions [31]. Reviews likewise report greater activation in frontal control regions such as the inferior frontal gyrus and anterior cingulate cortex when speech is degraded, even when intelligibility is maintained [26]. Together, these findings suggest that hearing loss may impose persistent cognitive demands in everyday listening, with possible downstream effects on cognitive reserve and resource allocation over time [27,32].

### 4.2. Sensory Deprivation and Neuroplasticity

A second mechanism proposes that sensory deprivation and neuroplastic changes may result from reduced auditory input. Chronic auditory deprivation may lead to structural and functional alterations in the brain, particularly in the auditory cortex and related temporal regions.

Neuroimaging studies report associations between hearing loss and reduced gray matter volume in auditory cortex as well as altered large-scale functional connectivity [32]. Reviews of structural and functional imaging studies similarly describe changes in auditory cortex structure, altered activation during speech perception, and increased recruitment of frontal control networks in individuals with hearing impairment [33,34]. Prolonged hearing loss may also lead to cross-modal plasticity, in which visual or multisensory processing increasingly recruits auditory cortical regions [35]. Such reorganization has been observed even in mild hearing loss and may reflect compensatory neural adaptation to reduced sensory input [36]. Importantly, structural changes associated with hearing loss extend beyond the primary auditory cortex. Meta-analytic evidence indicates alterations in frontal regions and frontotemporal white matter pathways, suggesting broader effects on distributed neural networks involved in cognitive control and attention [32]. Experimental animal studies further support this possibility, with evidence that noise-induced hearing loss may also affect hippocampal neurogenesis, pointing toward potential downstream effects on brain regions involved in memory and plasticity [37,38,39].

### 4.3. Social Isolation and Psychosocial Pathways

Hearing loss may also influence cognitive aging through indirect psychosocial pathways. The relative importance of these pathways may vary across the life course, and in later life, consequences of hearing loss such as social withdrawal, loneliness, and depression may represent particularly relevant contributors to cognitive decline. Impaired hearing not only affects communication. It also increases risk of social withdrawal, loneliness, and depressive symptoms, factors that are themselves associated with cognitive decline and dementia. Studies suggest that part of the association between hearing loss and cognitive outcomes may be explained by the relationship with these psychosocial factors [9,40]. For example, longitudinal data from the English Longitudinal Study of Ageing indicate that loneliness and social isolation partly account for the link between hearing impairment and decline in episodic memory [41]. A recent meta-analysis reported a modest but significant mediating role (i.e., on the path between hearing loss as an exposure which then in turn leads to cognitive impairment) of social engagement in the association between hearing loss and cognitive decline, particularly among individuals with lower levels of social participation [42]. Depressive symptoms may play a related role. Some studies indicate that the relationship between hearing loss, depression, and cognitive impairment is influenced by the quality of social relationships [43]. At the same time, evidence remains mixed regarding the extent to which psychosocial factors meaningfully explain sensory–cognitive associations. For example, a large population-based study from the Canadian Longitudinal Study on Aging found that although social variables were independently associated with both sensory and cognitive functioning, their mediating and moderating effects on sensory–cognitive associations were generally weak and mostly non-significant [7]. Together, these findings suggest that psychosocial factors may contribute to the link between hearing impairment and cognitive decline in some individuals or contexts, but are unlikely to fully account for the association.

### 4.4. Shared Neuropathology and Vascular Mechanisms

An alternative explanation is that hearing loss and cognitive decline share common biological mechanisms, rather than reflecting a direct causal relationship. Rather than a direct or indirect relationship through a different condition, both conditions may arise from overlapping pathological processes affecting the auditory and central nervous systems. Proposed shared mechanisms include microvascular disease, neuroinflammation, mitochondrial dysfunction, and oxidative stress, which may contribute to both cochlear degeneration and neurodegeneration [44,45]. There is also emerging evidence suggesting overlap with Alzheimer’s disease-related pathology itself, including findings of amyloid-β42 in cochlear perilymph, further supporting the possibility of shared neurodegenerative processes affecting both auditory and cognitive systems [46]. Cerebrovascular dysfunction may be particularly relevant, as microvascular pathology can affect both inner ear structures and brain regions involved in cognition [44]. Molecular processes of neurodegenerative diseases are not limited to classical memory-related brain regions but may also affect areas involved in the processing of auditory information [45]. Age-related changes to the auditory system are well-documented. However, how and if these changes stem from sources like overall neurodegeneration or microvascular disease remains unclear. From a clinical perspective, understanding the contribution of these common causes is important when evaluating the extent to which effective hearing-loss management may reduce the risk of dementia. If common causes such as those described here contribute substantially to the overall hearing–dementia relationship, management of hearing loss itself may have a more limited direct effect on dementia risk reduction, while still potentially reducing risk indirectly through socially mediated pathways.

Importantly, hearing loss may function both as a modifiable exposure and as a marker of broader biological vulnerability [47]. For clinicians, this implies that identifying hearing impairment may have dual relevance: it may point to an opportunity for intervention, but it may also warrant increased awareness of potential underlying neurodegenerative disease relevant for interprofessional discussions.

### 4.5. Reverse Causation and Early Neurodegenerative Changes

Reverse causation has been proposed as a potential explanation for the association between hearing loss and dementia. Changes in peripheral hearing and pure tone thresholds have been suggested to lead to changes in cognitive function in older adults; however, evidence remains limited. Consistent with this, deficits in complex listening tasks (e.g., speech-in-noise perception, dichotic listening, temporal processing) have been reported in individuals with mild cognitive impairment and early Alzheimer’s disease [48]. Some studies further suggest links between central auditory dysfunction and Alzheimer-related biomarkers, including cerebrospinal fluid tau, cortical thickness, and hippocampal volume [49,50]. However, the extent to which these findings reflect early manifestations of neurodegenerative disease rather than independent risk processes remains unclear.

## 5. Clinical Intersections

### 5.1. Hearing Loss in Cognitive Testing

In research and clinical settings, dementia adjudication and diagnosis incorporate patient/family case history, medical imaging, and neuropsychiatric testing across a variety of domains. It is possible unrecognized hearing impairment may artificially reduce cognitive test performance, particularly in assessments relying on verbally delivered instructions or test modality. Widely used cognitive screening tools such as the Mini-Mental State Examination (MMSE) and Montreal Cognitive Assessment (MoCA) rely heavily on auditory comprehension through a verbally administered screener [51,52]. Empirical studies demonstrate that hearing loss can significantly influence cognitive test scores when appropriate awareness of hearing and accommodations are not heeded and hearing difficulty is more advanced. For example, older adults with hearing impairment performed substantially better on a written version of the MMSE compared with the standard verbally administered version, suggesting that auditory barriers can lead to underestimation of cognitive ability [53]. Similar findings have been reported for common cognitive screening tools, where hearing impairment was associated with poorer test scores [54]. When auditory input is artificially degraded to simulate age-related hearing loss, individuals with otherwise intact cognition show poorer performance on cognitive tasks [55]. At the same time, hearing–cognition associations are not limited to tasks with high auditory or perceptual demands. Associations between hearing loss and poorer cognitive performance have also been observed for predominantly non-auditory measures, including verbal fluency tasks with minimal perceptual load, suggesting that test-related auditory bias alone is unlikely to fully explain the observed relationship between hearing impairment and cognition [56,57]. This interpretation is further supported by recent evidence demonstrating associations between hearing loss and performance on non-auditory tasks, including measures of spatial orientation, indicating that the effects of hearing impairment may extend beyond speech perception and auditory processing [58].

Misunderstanding instructions or delayed auditory processing could contribute to misclassification of cognitive impairment in older adults [59], as performance is improved on visually administered cognitive tests for those with more significant hearing loss compared to cognitive tasks relying on auditory information [60]. Hearing screening prior to cognitive testing has therefore been recommended but remains inconsistently implemented in clinical practice. Additional recommendations include ensuring appropriate accessibility for cognitive testing (i.e., the older adult is wearing hearing aids if available or has use of assistive listening devices, verbal instructions are supplemented with written instructions). When administering any cognitive test battery, incorporating a mix of auditory and verbally administered tests may minimize misclassification of severity of cognitive difficulty by ensuring evaluation is not solely dependent on any one sensory domain.

### 5.2. Integrated Screening Approaches

Given the high prevalence of hearing impairment in older adults, helping other disciplines understand how brief hearing assessments may be incorporated into clinical practice may be valuable for geriatric and cognitive clinics for comprehensive patient evaluation, including understanding the distinctions between screening as simple audiometric measures and validated self-report instruments. Although self-reported hearing difficulty may not comprehensively capture all forms of hearing impairment, older individuals who report hearing problems are nevertheless likely to experience clinically meaningful deficits [61]. These findings suggest inclusion of auditory processing measures and audiometric measures (screening or pure-tone average [PTA]) could complement traditional cognitive screening in identifying individuals at elevated risk. This inclusion is also important for ensuring perceived symptoms are appropriately attributed to cognitive decline rather than impaired hearing. Even brief objective screening or self-reported hearing assessment may serve as an important first step in a broader referral pathway for more comprehensive audiological and cognitive evaluation when indicated. Furthermore, integrating audiological screening into cognitive assessments—and cognitive screening into audiological assessments—may improve diagnostic accuracy, reduce hearing-related bias in cognitive testing, and support the development of individualized management and rehabilitation strategies tailored to an older adult’s cognitive and auditory needs [62,63].

### 5.3. Diagnostic Challenges

Differentiating between peripheral hearing loss, central auditory processing deficits, and early dementia-related auditory dysfunction can be challenging in clinical practice, especially in the context of cognitive decline. Central auditory processing disorder (CAPD), in particular, may present symptoms that overlap with both peripheral hearing loss and early cognitive impairment, resulting in possible complications during assessments and resulting diagnoses. Central auditory deficits may be more strongly associated with future dementia risk than peripheral hearing loss alone [64]. In older adults undergoing comprehensive audiological and neuropsychological assessment, measures of central auditory dysfunction, such as performance on competing speech tasks, have shown stronger associations with Alzheimer’s disease than pure-tone hearing thresholds [65].

These findings underscore the need for careful interpretation of auditory symptoms in older adults and highlight the importance of integrated assessment approaches that consider both sensory and cognitive factors with interprofessional collaboration essential for holistic and person-centered diagnosis. More broadly, these observations highlight the value of interdisciplinary collaboration among audiologists, neurologists, geriatricians, family physicians, nurse practitioners, and psychologists. Integrating hearing screening into cognitive evaluation may improve diagnostic accuracy and facilitate earlier identification of individuals who may benefit from auditory rehabilitation or further neurological assessment [55]. Further, detailed audiologic reports, including characterization of hearing loss severity and patterns as well as identification of atypical or clinically meaningful findings, should be shared across healthcare teams together with clear rehabilitation plans to improve accessibility, interdisciplinary communication, and awareness.

### 5.4. Hearing Health Within Healthy Aging

Regardless of its potential role in cognitive decline and dementia, untreated hearing loss is associated with many functional aspects which contribute to not only dementia risk but also overall health in aging. These consequences, including social engagement, increased risk of loneliness and depression, and mobility limitations, among others, may contribute to functional decline and reduced independence in older adults. Addressing hearing loss should therefore support not only cognitive health but also sustained engagement in social, physical, and community activities. Compared to some late-life mental health- or dementia-focused interventions, attention to and management of hearing loss is a low-risk and comparatively minimal cost approach that can significantly enhance management tools for older adults [66]. Framing hearing care within healthy aging initiatives may help integrate sensory health into broader strategies aimed at maintaining well-being and functional capacity across later life. It also supports more balanced communication of research findings to clinicians, patients, and the public, helping to avoid overly alarmist messaging that frames hearing management as necessary to prevent dementia without sufficient evidential nuance. Regardless, substantial evidence supports the importance of hearing health across a range of outcomes relevant to older adults, extending beyond dementia itself. Hearing remediation may also help improve other potentially modifiable risk factors implicated in dementia prevention frameworks, including social isolation, depression, and physical inactivity [4].

## 6. Hearing Rehabilitation and Cognitive Outcomes

### 6.1. Observational Evidence

**Figure 2:**
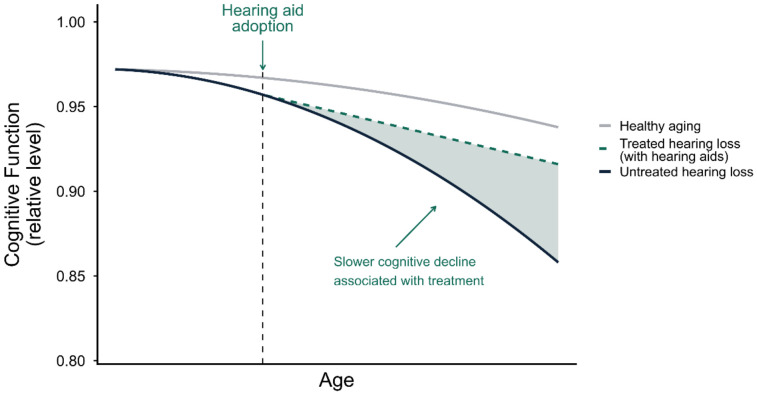
Hypothetical illustration of the potential impact of hearing loss treatment on cognitive decline trajectories. Research suggests that treating hearing loss may slow cognitive decline relative to untreated hearing loss, although outcomes may remain poorer than in individuals with normal hearing. The trajectories shown are conceptual and intended for illustrative purposes only; they are not based on observed data, statistical analyses, or fitted statistical models.

Across multiple cohort studies, as well as systematic reviews and meta-analyses, hearing rehabilitation has been associated with a lower risk of mild cognitive impairment and slower rates of cognitive decline among individuals with hearing loss compared to those who do not engage in treatment or rehabilitation (i.e., hearing aids, listening devices, counseling and education) [67,68,69]. This could be due to not only altering the functional consequences of hearing loss but also decreasing social withdrawal and depressive symptoms [70], both of which have been linked to cognitive decline [71]. Importantly, while individuals receiving hearing interventions tend to show more favorable cognitive trajectories than those with untreated hearing loss, their cognitive performance often remains lower than that of individuals with normal hearing (Figure 2), suggesting possibly earlier or continued underlying risk for cognitive decline that hearing intervention has not yet addressed in existing studies [72].

However, these findings should be interpreted with caution. Most of the available evidence comes from studies that compare people who use hearing aids with those who do not, rather than from randomized trials. Older adults who pursue and adopt hearing aids differ in many important ways from those who do not. As a result, the groups being compared may differ in important ways beyond hearing aid use. For example, individuals who obtain hearing aids often have higher levels of education, healthier lifestyles, better access to healthcare, and higher socioeconomic resources, despite having fewer auditory impairments [73]. These factors are themselves associated with a lower risk of cognitive decline, making it difficult to determine how much of the observed benefit is due to hearing aids (and associated variability in actual utilization of the device) versus these underlying differences. In addition, many studies have relatively short follow-up periods, which may limit the ability to detect long-term cognitive effects [67,68]. From a clinical perspective, this means that potential cognitive benefits of hearing interventions may not be captured within the time frame, utilization or context captured in most studies, as changes in cognitive trajectories may take several years to become apparent.

### 6.2. Randomized Controlled Trial Evidence

Randomized controlled trials have begun to address this research question and observational study limitations in a more controlled environment to provide more direct evidence of the influence of hearing intervention on dementia risk or cognitive change. The largest trial to date is the Aging and Cognitive Health Evaluation in Elders (ACHIEVE) study, which evaluated whether hearing intervention could influence cognitive decline in older adults with untreated hearing loss [74]. In this randomized trial, participants aged 70–84 years were assigned either to a hearing intervention, including audiological counseling and hearing aids, or to a health education control condition. Overall cognitive decline over three years did not differ significantly between groups in the full sample. However, among participants with higher baseline risk for cognitive decline, specifically those in the Atherosclerosis Risk in Communities (ARIC) cohort, who were generally older, more likely to be Black, had fewer years of education and lower income, exhibited higher rates of hypertension and diabetes, and had lower MMSE scores than participants in the other cohort, hearing intervention was associated with a 48% reduction in cognitive decline over the 3-year follow-up period compared to a healthy aging intervention [74], a significant improvement compared to other non-invasive interventions for cognitive decline currently available.

Although the trial did not demonstrate universal cognitive benefits of hearing intervention, the findings suggest that hearing rehabilitation may help mitigate cognitive decline in individuals at elevated risk and that follow-up is continuing to determine potential longer-term effects. Additional smaller trials and intervention studies have also explored cognitive outcomes following hearing rehabilitation, though results remain heterogeneous and follow-up periods are often limited [68].

### 6.3. Clinical Implications for Healthy Aging

Regardless of the magnitude of cognitive effects, hearing rehabilitation provides well-established benefits for communication, quality of life, and social participation.

Beyond impacts on communication, hearing rehabilitation may reduce depressive symptoms and improve psychosocial well-being in individuals with hearing loss [70]. Evidence regarding effects on social isolation and loneliness remains mixed [75]. Nonetheless, improvements in communication and daily functioning are consistently observed following hearing intervention [76]. Recent guidelines for age-related hearing loss outline related recommendations related to communication, safety, overall health and function, cognition, and quality of life, including how to counsel on treatment recommendations and referral adherence relevant for both patients and other health professionals. Importantly, recommendations should frame these points not as a prevention of decline but as promotion of maintained function, independence, and resilience.

Taken together, current evidence indicates that hearing rehabilitation offers substantial benefits for quality of life and social engagement in older adults with hearing loss. While effects on cognitive decline remain an active area of investigation, addressing hearing impairment represents an important component of comprehensive care for aging populations.

## 7. Overlap with Other Sensory Impairments

Hearing loss rarely occurs in isolation in later life. Age-related declines in vision, olfaction, and other sensory systems frequently co-occur, possibly reflecting shared biological vulnerabilities and cumulative exposure to systemic risk factors affecting neural and vascular health [77,78]. Increasing evidence suggests that multisensory impairment confers greater risk for cognitive decline and dementia than single-sense deficits [79]. Longitudinal studies indicate that combined sensory losses are associated with steeper cognitive decline than single-modality impairments. For example, individuals with dual hearing and vision loss show faster cognitive decline compared with those with normal sensory function or hearing loss alone [80,81]. Concurrent impairments in hearing, vision, and olfaction may reflect broader processes of brain aging, vascular, or neurodegenerative processes [77,82].

From a clinical perspective, the co-occurrence of sensory impairments may signal a higher-risk subgroup. Identifying more than one sensory deficit should therefore prompt increased attention to cognitive monitoring and broader risk assessment. It also highlights the importance of assessing multiple sensory systems in clinical practice, rather than focusing on hearing alone, as combined impairments may have greater implications for cognitive health and functional outcomes.

## 8. Future Directions

Several priorities should guide future research in this field. From a clinical perspective, a key unresolved issue is how to tease apart the contribution of hearing impairment across different parts of the auditory pathway, supported by clearer phenotyping and more consistent measurement across studies. This distinction is directly relevant for clinical interpretation. Peripheral hearing loss may represent a modifiable target for intervention through hearing aids to improve detection of auditory information; however, hearing aids do little to support restoration of speech perception when central auditory dysfunction may reflect early neurodegenerative changes. At present, it remains challenging to distinguish between these possibilities, making it difficult to determine whether hearing loss in a given individual should primarily be interpreted as a treatable condition, an early warning sign of cognitive decline, or both. However, this distinction may ultimately be of limited practical importance if the primary goal is to support functioning, communication, and quality of life in older adults.

Second, further longitudinal studies are needed to better understand the pathways linking hearing loss and cognitive decline, including the relative contributions of causal effects and shared biological vulnerability. Studies combining repeated auditory assessments with cognitive outcomes, neuroimaging, and biomarker data may help clarify when hearing loss becomes most relevant for cognitive change and which mechanisms are most closely linked to dementia-related processes. These associations should not only be investigated with the pure-tone audiogram, but also using naturalistic speech comprehension tests, such as speech-in-noise tasks, to more accurately reflect real-life auditory processing demands. While distinguishing between these pathways is important for refining clinical interpretation and targeting interventions, it is less critical for initial clinical action. Regardless of whether hearing loss acts as a causal factor or reflects underlying neurodegenerative processes, maintaining hearing health remains relevant for supporting healthy aging and should be addressed in clinical care.

Third, hearing measures should be more systematically evaluated for inclusion in dementia risk prediction models. This includes not only peripheral hearing assessments but also central auditory processing measures, which may prove especially informative in identifying early neurodegenerative change.

Fourth, more implementation research is needed to determine how hearing assessments can be embedded into geriatric, neurological, and memory clinic settings in ways that are feasible, acceptable, and clinically useful. This includes identifying which screening tools are most appropriate, who should administer them, and how abnormal findings should be managed. It will also be important to evaluate how hearing assessment and intervention can be incorporated into current multimodal dementia risk-reduction initiatives, such as FINGER, US POINTER, and related international prevention efforts, where sensory health may represent an important but still underrecognized component of maintaining cognitive functioning in later life [83,84].

Importantly, questions about how to provide hearing care and rehabilitation, what adaptations are most impactful, how to measure this effectiveness, and how to maintain focus on person-centered care remain unanswered. Current practices are largely driven by professional experience rather than evidence-based practices, creating significant heterogeneity in patient and family experiences.

## 9. Conclusions

The intersection between hearing loss and cognitive decline represents an important area of aging research and clinical care. Epidemiological studies consistently support an association between hearing impairment, cognitive decline, and dementia, and several plausible mechanisms may underlie this relationship, including increased cognitive load, sensory deprivation, psychosocial pathways, shared neuropathology, and early neurodegenerative changes affecting central auditory processing.

Although causality is not yet fully established, hearing impairment is common, measurable, and treatable. For that reason alone, it deserves greater attention in clinical settings caring for older adults. At a minimum, hearing should be considered when interpreting cognitive test performance and when evaluating communication difficulties in individuals with cognitive concerns. More broadly, hearing health may represent an important component of strategies aimed at supporting cognitive function, communication, participation, and healthy aging.

Integrating hearing awareness into geriatric, family, and cognitive practice is therefore a pragmatic step, even as the field continues to refine the evidence base regarding dementia prevention and cognitive outcomes.

## Figures and Tables

**Table 1:** Summary of large-scale epidemiological studies on hearing loss and dementia.

Authors	Study Design	Cohort (Country)	*n*	Conclusion
Liang et al., 2021 [14]	Meta-analysis (Longitudinal)	14 cohorts (Australia: 1; Denmark: 1; UK: 1; USA: 10)	726,900	Hearing loss associated with dementia (HR 1.59, 95% CI: 1.37–1.86) and Alzheimer’s disease (HR 2.24, 95% CI: 1.32–3.79).
Loughrey et al., 2018 [8]	Meta-analysis (Cross-sectional & Longitudinal)	35 cross-sectional studies (Australia: 9; Europe *: 14; Japan: 1; North America ^†^: 11) 11 cohorts (Australia: 4; Europe ^‡^: 3; Japan: 2; North America ^§^: 2)	Cross-sectional: 15,260 Longitudinal: 8233	Cross-sectional: cognitive impairment (OR 2.00), dementia (OR 2.42); longitudinal: cognitive impairment (OR 1.22), dementia (OR 1.28), not AD (OR 1.69, non-significant).
Wei et al., 2017 [13]	Meta-analysis (Longitudinal)	10 cohorts (Singapore: 1; UK: 1; USA: 8)	15,521	Hearing impairment associated with MCI (RR 1.30, 95% CI: 1.12–1.51) and dementia (RR 2.39, 95% CI: 1.58–3.61).
Yu et al., 2024 [15]	Meta-analysis (Longitudinal)	50 cohorts (Australia: 5; East Asia ^¶^: 11; Europe ^‖^: 10; USA: 24)	1,548,754	Hearing loss associated with dementia (HR 1.35, 95% CI: 1.26–1.45), MCI (HR 1.29, 95% CI: 1.11–1.50), cognitive decline (HR 1.29, 95% CI: 1.17–1.42), AD (HR 1.56, 95% CI: 1.30–1.87), not vascular dementia (HR 1.30, 95% CI: 0.83–2.05); +16% risk per 10 dB.
Zhao et al., 2025 [10]	Cross-sectional & Longitudinal	China Health and Retirement Longitudinal Study (CHARLS; China)	Cross-sectional: 7891 Longitudinal: 5326	Cross-sectional: cognitive impairment (OR 1.76); longitudinal: risk (HR 1.24), stronger for moderate–severe (HR 1.32); mediation by depression (20.83%) and social isolation (4.17%).

Note. Values are adjusted for covariates where reported. Sample sizes reflect analysis-specific samples and may overlap across study designs. * Denmark, Finland, and Sweden: 1; Finland: 1; Germany: 4; Italy: 1; Spain: 1; The Netherlands: 4; United Kingdom: 2. ^†^ Canada: 1; United States: 10. ^‡^ Germany: 1; The Netherlands: 1; United Kingdom: 1. ^§^ Canada: 1; United States: 1. ^¶^ China: 2; Korea: 2; Japan: 3; Singapore: 1; Taiwan: 3. ^‖^ Denmark: 1; France: 1; Germany: 3; Italy: 1; Norway: 1; Sweden: 1; United Kingdom: 2.

## Data Availability

No new data were created or analyzed in this study. Data sharing is not applicable to this article.

## Abbreviations

The following abbreviations are used in this manuscript:

MMSE	Mini-Mental State Examination
MoCA	Montreal Cognitive Assessment
AD	Alzheimer’s disease
